# Flexible Intramedullary Nails for Stabilization of Transverse Femoral Fractures in Cats: Ex Vivo Biomechanical Characterization

**DOI:** 10.3390/ani16081154

**Published:** 2026-04-10

**Authors:** Rebeca Bastos Abibe, Sheila Canevese Rahal, René Quispé Rodriguez, Guilherme Rech Cassanego, Fátima Maria Caetano Caldeira, Philipp Kobbe, Jörg Eschweiler, Luis Fernando Nicolini

**Affiliations:** 1 Department for Trauma and Reconstructive Surgery, University Hospital Halle (Saale), Ernst-Grube-Straβe 40, 06120 Halle (Saale), Germany; philipp.kobbe@uk-halle.de (P.K.); joerg.eschweiler@uk-halle.de (J.E.); 2 Department for Trauma and Reconstructive Surgery, BG Hospital Bergmannstrost Halle (Saale), Merseburger Straβe 165, 06112 Halle (Saale), Germany; 3 School of Veterinary Medicine (FMVZ), Sao Paulo State University (UNESP), Prof. Doutor Walter Mauricio Correa s/n, Botucatu 18618-681, Brazil; sheila.canevese-rahal@unesp.br (S.C.R.); guilherme.rech@unesp.br (G.R.C.); 4 Department of Mechanical Engineer, Santa Maria Federal University (UFSM), Roraima Av. 1000, Santa Maria 97105-340, Brazil; rene.rodriguez@ufsm.br (R.Q.R.); nicolini.luis@ufsm.br (L.F.N.); 5 Department of Surgery, University of Sao Paulo (USP), Prof. Dr. Orlando Marques de Paiva 87 St., Sao Paulo 05508-270, Brazil; fatimacaldeira@usp.br

**Keywords:** FIN, biomechanical testing, fracture

## Abstract

Flexible intramedullary nails (FINs) are commonly used in children and adolescents to treat long bone fractures, but few studies exist in animals. This study aimed to evaluate the biomechanical performance of FINS for the stabilization of transverse femoral fractures in cats. Fifteen bones were kept intact, while in another 15 bones, a mid-diaphyseal transverse fracture was induced and stabilized with two steel FINs of equal diameter, advanced divergently toward the greater trochanter and femoral neck, with end caps applied to the free ends. Five constructs and five intact bones were subjected to axial compression, four-point bending, and torsion tests. In axial compression, intact bones showed higher mean maximum force (1090.51 N vs. 608.43 N) and stiffness (845.98 vs. 298.86 N/m) than constructs. In bending, intact bones reached a maximum force of 1384.75 N, whereas a distinct maximum force could not be determined for the constructs; stiffness was also greater (1580.92 vs. 13.32 N/m). In torsion, intact bones demonstrated substantially higher mean maximum force (6.764 vs. 0.166 Nm) and stiffness (32.11 vs. 1.04 Nm/rad) than constructs. In conclusion, FINs with end caps demonstrate low construct stiffness, particularly under torsional loads, when used to stabilize mid-diaphyseal transverse femoral fractures in cats.

## Simple Summary

Flexible intramedullary nails are commonly used to stabilize long bone fractures, particularly in children, but their biomechanical performance in cats is not well understood. Biomechanical studies are important because they allow researchers to evaluate how a stabilization method behaves before it is applied in live animals. In this ex vivo study, intact femoral bones from adult cats were compared with bones that were fractured and stabilized using flexible intramedullary nails with end caps. The bones were tested under compression, bending, and torsion to assess their strength and stiffness. Intact bones demonstrated significantly greater strength and stiffness than those stabilized with flexible intramedullary nails in all loading conditions, with the largest differences observed under torsional forces. These findings indicate that flexible intramedullary nails can be an option for treating certain femoral fractures in cats, but their mechanical strength is lower than that of intact bone and should be taken into consideration. Understanding these limitations can help veterinarians select the most appropriate treatment and improve recovery outcomes for injured cats.

## 1. Introduction

Several methods have been used to stabilize femoral fractures in cats, which are among the most common long bone fractures in this species [1,2]. The choice of fixation method can be based on three fracture score areas: mechanical or biomechanical, biological, and clinical [1,3]. Some characteristics of cats also need to be considered, such as their propensity to climb and jump, and those that exhibit intractable and uncooperative behavior [4]. The feline femur is suitable for the use of intramedullary implants due to its straight tubular shape with a large medullary canal, but care must be taken to avoid iatrogenic injury to the sciatic nerve if the implants are positioned near the greater trochanter [1,3,4].

Flexible intramedullary nails, developed in the early 1980s by the Nancy group in France, are a load-sharing technique that provides reliable fixation by maintaining relative stability and promoting indirect fracture healing, while also being cost-effective and easy to insert and remove [5]. The method is commonly used in children and adolescents to treat long bone fractures, such as the femur, providing stable but elastic fixation [6,7]. Flexible intramedullary nails are particularly applicable to diaphyseal femoral fractures with a transverse or short oblique configuration [6].

At least two flexible intramedullary nails are introduced through the metaphysis into the medullary canal and advanced to the opposite metaphysis [7]. These nails can withstand angular and compression forces [8]. Flexible intramedullary nails are typically made of stainless steel or titanium alloys, with ongoing debate on the optimal composition [6,8]. Furthermore, characteristics such as tip shape [8] and interlocking systems, such as caps or screws, vary according to the commercial system [9,10].

Biomechanical studies have tested flexible intramedullary nails on canine bones (femur, radius, ulna, and tibia) [11,12], and ovine and lamb tibias [9,10]. Clinical studies have also used this method in tibia or radius/ulna fractures in dogs [13,14]. To the author’s knowledge, this technique has not yet been evaluated in feline models. Therefore, the aim of this study was to assess the biomechanical performance of flexible steel intramedullary nails for the stabilization of transverse femoral fractures in cats. The monotonic compression, bending and torsional loading properties of flexible steel intramedullary nail constructs were investigated and characterized. These properties were also investigated for intact femurs as a reference.

## 2. Materials and Methods

The Institutional Ethics Committee approved this study for the Use of Animals (000.209-CEUA).

### 2.1. Harvesting and Preparation of the Specimens

Thirty femurs were obtained from adult domestic cats (mean age 4.4 ± 1.0 years; mean body mass 4.5 ± 1.3 kg), including nine females and six males, that died or were euthanized for reasons unrelated to this study. The sample size was defined pragmatically, given specimen availability and the experimental nature of the study. After removing all soft tissues from the femurs, the specimens were wrapped in gauze moistened with 0.9% saline solution, placed in numbered plastic bags, and stored at −20 °C. At the time of mechanical testing, the bones were thawed at room temperature. Each bone was radiographed (digital equipment SIUI SR-8100, Shantou Institute of Ultrasonic Instruments Co., Ltd., Shantou, China) to rule out bone lesions. Bones with radiographic evidence of lesions or abnormalities were excluded. All specimens were numbered, and the allocation list was generated using QuickCalcs (GraphPad Software) (version 11.0.0), which shuffled the sample order. Fifteen bones were kept intact, while 15 bones were fractured and stabilized with intramedullary nails.

**Figure 1:**
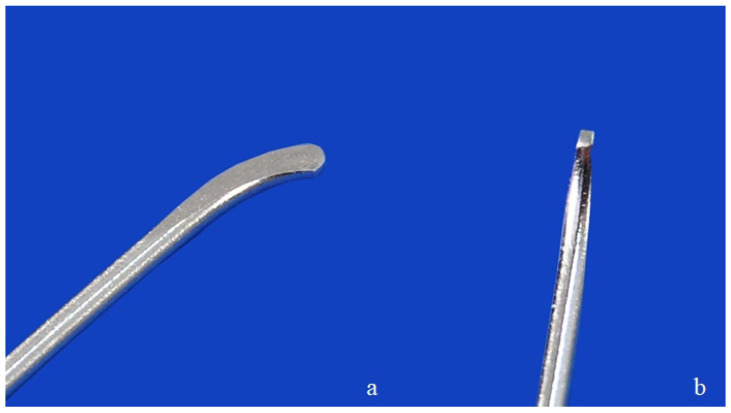
Tip of the stainless steel flexible intramedullary nail, illustrating the pre-curved segment (**a**) and the smoothly rounded tip (**b**) designed to facilitate insertion.

**Figure 2:**
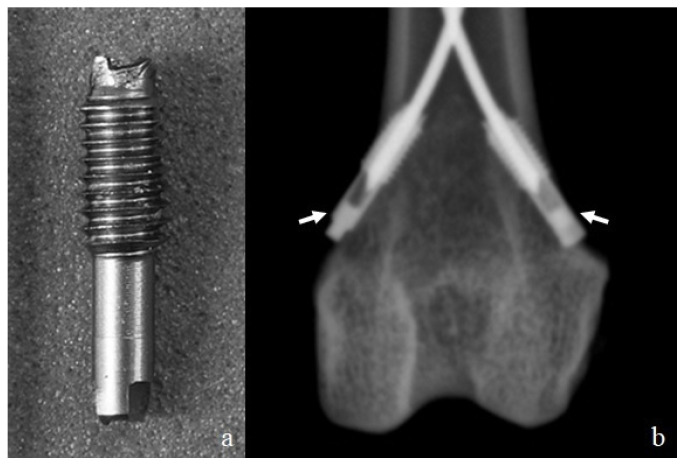
(**a**) Model of the end cap. (**b**) Craniocaudal radiograph of a feline distal femur showing the two end caps (arrows) inserted into the cortical bone.

**Figure 3:**
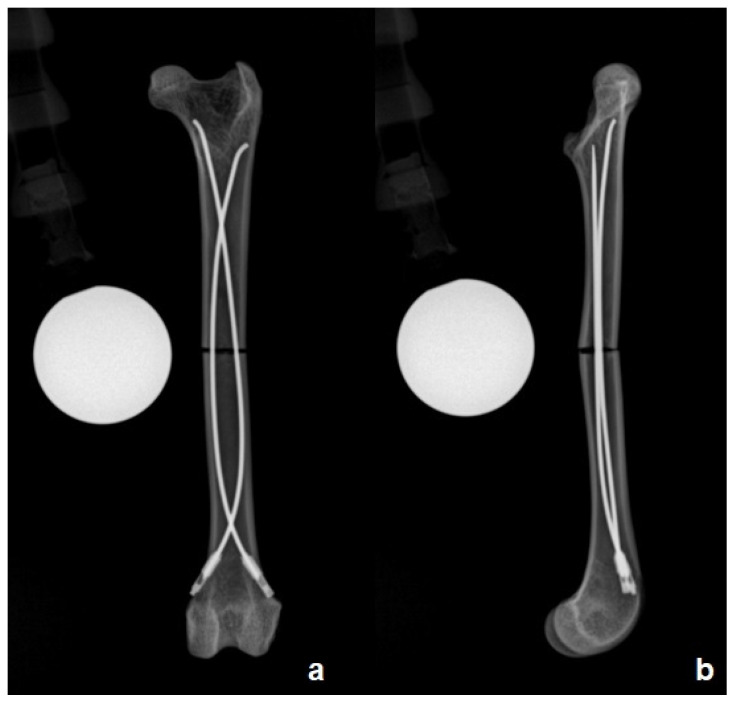
Craniocaudal (**a**) and mediolateral (**b**) radiographic views of a feline femur, including calibration spheres, demonstrating a mid-diaphyseal transverse fracture stabilized with two flexible stainless-steel intramedullary nails. The nails are appropriately positioned within the medullary canal, providing alignment and stabilization of the fracture fragments, with one nail directed toward the greater trochanter region and the other toward the femoral neck region. Note the end caps placed on the nail tips and secured into the cortical bone.

To prepare for the use of the intramedullary nails, the bone length was measured from the femoral head to the condyles’ surface, and the midpoint of the diaphysis was determined. A mid-diaphyseal transverse fracture was induced with an oscillating saw. The fracture was stabilized with two steel flexible intramedullary nails of equal diameter. The passivated stainless steel flexible nails (ASTM F138) [15] had a diameter of 1 mm and a length of 100 mm with a curved tip (Aldrivet, Campinas, Brazil) (Figure 1). The flexible nails were inserted both medially and laterally from the supracondylar region of the femur. The entry point was marked with a 2 mm Steinmann pin perpendicular to the bone (90° to the longitudinal axis), which was gradually tilted to facilitate oblique insertion. The first flexible nail (medial) was introduced into the entry hole with the angled tip positioned toward the medullary canal. Next, the second flexible nail (lateral) was introduced in the same manner. The flexible nails were inserted using a Jacobs chuck with a handle, advancing them gradually with alternating half-turns clockwise and counterclockwise until they reached the vicinity of the fracture site. The fracture was reduced, and the nails were advanced across the fracture site to the proximal metaphysis. One nail was directed towards the greater trochanter region and the other towards the femoral neck region. The same operator inserted all the flexible intramedullary nails. Radiographs were taken to ensure the correct placement of the flexible nails. Subsequently, the free ends of the nails were cut, and protective end caps (Length: 9.5 mm; diameter: 2.3 mm with threads, 1.5 mm without threads) (Aldrivet, Campinas, Brazil) (Figure 2a) were placed over the exposed ends. These end caps were then threaded clockwise into the bone at the entry site (Figure 2b). The final construct of the flexible intramedullary nails exhibited reciprocal curvature, with the apex of each nail positioned at the mid-diaphyseal region of the femur, ensuring stable divergent fixation across the fracture site. Radiographs of the constructs were taken in craniocaudal and mediolateral views (Figure 3).

### 2.2. Static Mechanical Testing

Each test, such as axial compression with eccentric load, four-point bending, and torsion, was conducted on five constructs and five intact bones. The constructs were tested to failure to determine the maximum force. For constructs, failure was defined as deformation of the flexible nails, which would result in bone misalignment in a clinical situation, and/or bone breakage. Additionally, mechanical failure was defined as the loss of the material’s ability to withstand loads, observed as a drop in the load–displacement curve. For intact bones, testing was continued until failure (i.e., fracture accompanied by a sharp drop in load). Intact bones were used to establish baseline values for the maximum force in each test. Biomechanical tests were conducted in a climate-controlled environment at a temperature of 21 ± 2 °C.

For axial testing with eccentric load, the distal end of the femur was potted in polymethylmethacrylate resin. The femur was kept perpendicular, and the distance from the femoral head to the acrylic resin base was maintained constant (60 mm). The axial load was applied through the femoral head at a speed of 5 mm/minute using a universal testing machine (Shimadzu AG-IC 100 kN-Shimadzu Corporation, Kyoto, Japan) with a 100 kN load cell. Since the static axial compression test was conducted with an eccentric load, two phenomena occurred simultaneously: compression and bending. Therefore, it is crucial to consider the impact of load eccentricity when associating the applied force with the displacement. The recorded parameters were force and displacement, measured directly from the testing machine.

The four-point bending test was conducted on the femur in the sagittal plane. The femur was positioned horizontally with the caudal surface facing up. The cranial surface was supported between two rollers spaced 50 mm apart. The upper rollers were spaced 20 mm apart, with the fracture site centered in the middle. All rollers were 10 mm in diameter. The vertical actuator speed was set at 5 mm/min. The test followed ASTM F1264-A1 [16] guidelines. The recorded parameters included force and displacement, acquired directly from the testing machine. The test was stopped upon detection of a sudden drop in load in intact bones. In the constructs, there was no abrupt reduction in the applied load. The test was manually stopped after reaching a minimum displacement of 5 mm. Due to variability in the experimental response among specimens, a specific deformation region for stiffness calculation could not be universally defined. Therefore, stiffness was calculated from the initial linear portion of the force–displacement curve. This linear range was selected where the coefficient of determination (R^2^) of the linear fit exceeded 0.9.

Torsion testing was conducted using a torsion test machine (MOOG Model G-414-804, Moog Inc., Elma, NY, USA) with a torque capacity of 50 Nm. The resin-fixed end of the construct was rotated clockwise at a speed of 1 rpm. The distance was maintained at 40 mm. The test was based on ASTM F1264-A2 [16] standards.

The torsion stiffness, *k_t_*, can be determined by the following expression:

(1) $$k_{t}=\frac{T}{\theta }$$

where T is the applied torque, and θ is the twisting angle. The stiffness was calculated using a linear fit considering the torque and angle data. In addition, the initial stiffness reduction in the constructs was visually assessed.

Stiffness (P/δ) was defined as the slope of the linear region of the force–displacement curve, representing structural resistance to deformation under load. The linear region was identified using a minimum coefficient of determination (R^2^) of 0.9, a commonly adopted criterion in biomechanical testing to ensure that stiffness values reflect predominantly elastic mechanical behavior.

### 2.3. Statistical Analysis

Descriptive statistics were provided for the biomechanical testing data, including the mean, standard deviation, median, standard error, coefficient of variation, and quartiles. All analyses were performed using Minitab software—version 19.

Due to the exploratory biomechanical nature of the study and the limited sample size (*n* = 5 per test condition), no inferential statistical tests were performed. The analysis was restricted to descriptive statistics, aiming to characterize mechanical behavior patterns rather than to establish statistical significance between groups.

## 3. Results

The length of the femurs was 11.11 cm (±0.48 cm). During axial compression testing, bone breakage and deformity of flexible intramedullary nails occurred at the fracture site, while intact bones showed fractures in the femoral neck region. One construct was damaged during biomechanical testing and had to be replaced.

**Figure 4:**
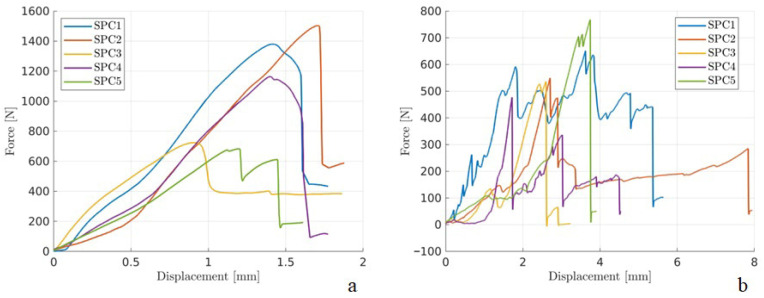
Graphs of force (N) versus displacement (mm) obtained in axial compression testing of feline femurs. (**a**) Intact femurs. (**b**) Feline femur constructs with mid-diaphyseal transverse fractures stabilized with two flexible stainless-steel nails. The straight line represents the region where the linear fit was made to determine stiffness, which is the slope of this line (**a**).

The maximum force values were 1090.51 N (±374.12 N) for the intact bones (Table 1) and 608.43 N (±101.2 N) for the constructs (Table 2). Figure 4 displays the force–displacement curves, showing dispersion in the compressive behavior for the intact femurs (Figure 4a) and constructs (Figure 4b). The stiffness values for intact femurs and constructs are presented in Table 3. Figure 4a illustrates the challenge of identifying consistent linear regions. When fitted linearly, these regions (manually selected) allow for obtaining the stiffness parameter (P/δ).

**Figure 5:**
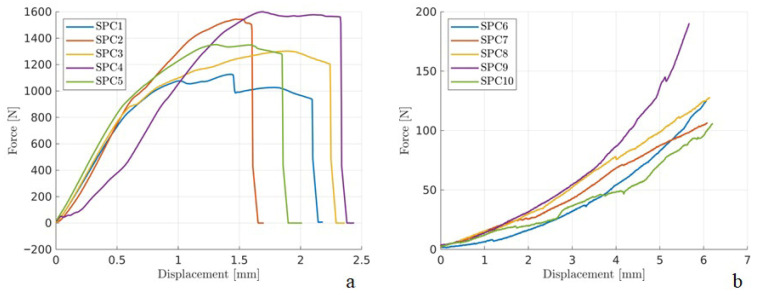
Graphs of force (N) versus displacement (mm) obtained from four-point bending tests of feline femurs. (**a**) Intact femurs. (**b**) Feline femur constructs with mid-diaphyseal transverse fractures stabilized with two flexible stainless-steel nails.

In the four-point bending test, failure of the constructs occurred due to deformity of flexible nails at the fracture site, while intact bones exhibited fractures in the femoral diaphysis. The maximum force values were 1384.75 N (±191.08 N) for the intact bones (Table 1). In the case of these constructs, there was no clear maximum force. Nonetheless, the monotonically increasing force indicates deformation, which may compromise stability and impair healing. The force–displacement curves are demonstrated in Figure 5, and the stiffness values for intact femurs and constructs are presented in Table 4.

**Figure 6:**
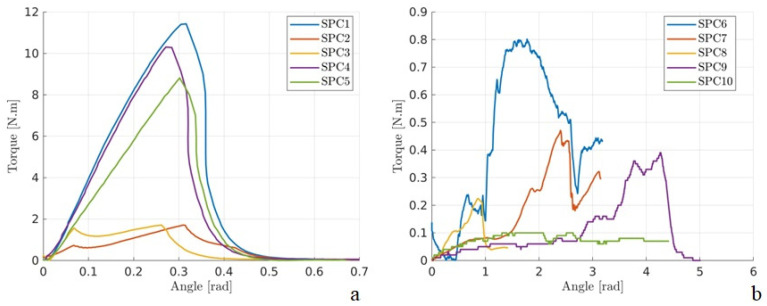
Graphs of torque (Nm) versus angular displacement (rad) obtained from torsion testing of feline femurs. (**a**) Intact femurs. (**b**) Feline femur constructs with mid-diaphyseal transverse fractures stabilized with two flexible stainless-steel nails.

Torsion testing resulted in failures in both constructs and intact bones due to bone breakage. The values were 6.764 Nm (±4.764 Nm) for the intact bones (Table 1) and 0.166 Nm (±0.075 Nm) for the constructs (Table 2). Figure 6 shows the force–displacement curves, and torsion stiffness values are shown in Table 5. In the case of specimen SPC9, the first linear region exhibited a large displacement of 3.54 rad, indicating that the calculated stiffness does not represent the material’s linear behavior in this instance.

## 4. Discussion

This in vitro study using feline femora observed that both stiffness and yield load were influenced by the type of mechanical test applied, in constructs stabilized with flexible intramedullary nails as well as in intact bones. Flexible intramedullary nails were evaluated for stabilization of a mid-diaphyseal transverse femoral fracture, as this fracture configuration represents a recognized indication for the technique in human medicine, particularly in children older than five years and in adolescents, among other treatment options [6]. Because fracture configuration can influence system stability, and long oblique or comminuted fractures are considered unstable [17], a transverse pattern was selected. In addition, the method was applied to adult specimens due to sample availability. This differs from its predominant clinical use in human orthopedics, where it is mainly indicated for skeletally immature patients [6,7].

Stainless steel flexible intramedullary nails were selected for compatibility with the size and mechanical demands of adult feline bones. Flexible nails are commercially available in stainless steel and titanium alloys [6,8]. Although debate persists regarding the optimal material, implant selection is often based on surgeon preference [8]. A systematic clinical review in pediatric femoral shaft fractures found no consistent evidence favoring titanium over stainless steel systems; however, a trend favored stainless steel due to lower implant cost [18]. Cost considerations are likewise relevant in veterinary practice and may influence implant selection. From a material standpoint, stainless steel exhibits higher tensile strength and elastic limit compared with titanium, potentially making it more suitable for patients with narrow medullary canals or greater body mass [8]. Flexible nails with a curved end were used and inserted without difficulty in the present study. Typically, flexible nails have beaked or hooked tips that facilitate sliding along the endosteal surface while minimizing cortical perforation, and their flattened outer surface eases insertion [8]. Additionally, blunt-ended designs are considered more suitable for diaphyseal fractures, since insertion of the nails into the medullary canal is relatively easy [7]. The standard technique for femoral shaft fractures recommends the use of two flexible intramedullary nails of similar thickness, with their combined diameter occupying 40% of the narrowest portion of the medullary canal [7,19]. The same procedure was performed in the current study, where the diameter occupied by the flexible nails was approximately 38.5% of the medullary canal on average, as determined by digital radiographic measurements with calibration [7].

Due to the small diameter of the flexible intramedullary nails, pre-contouring was not performed. In this approach, the nails become contoured during insertion [7]. Other authors recommend using flexible nails that are pre-curved before insertion [4,16]. This can be achieved by bending them beyond their elastic limit (from the tip to three times the diameter of the isthmus), so that they resist the tendency to straighten [8]. Clinically, protruding nail ends are often covered to protect the skin and facilitate later removal. Complications at insertion sites frequently involve skin irritation, infection, hematoma, seroma, or discomfort caused by implant prominence [6,20,21]. These complications may be mitigated by protective end caps, rounded nail ends, or burying the nail tip within the cortex [22]. Although not evaluated biomechanically in this study, end caps may also enhance construct stability, depending on the interlocking design [9,10]. Care must be taken to avoid overtightening, which may cause distraction at the fracture site.

Biomechanically, constructs stabilized with flexible nails showed lower mean stiffness and load-bearing capacity than the descriptive values for intact bones, an expected outcome with this fixation method and consistent with findings in pediatric synthetic femoral models [23]. In a study of growing beagle femurs, three-point bending testing showed that the mean load of the flexible nail system was approximately 40% of the initial value of the load-bearing capacity of intact bones [11]. The relatively low stiffness values observed in this study reflect the biomechanical principle of flexible nailing, which provides stability through elastic three-point fixation and controlled micromotion at the fracture site [6,8].

The biomechanical behavior observed in the present study may also be interpreted in light of concepts familiar to veterinary orthopedics, such as the “Rush pin effect,” in which stability is achieved through elastic interaction between the intramedullary implant and the endosteal surface [24,25,26]. Although the implants evaluated here differ in geometry and degree of standardization, both approaches rely on the principle of relative stability and controlled deformation under load [17,24,27,28]. However, Elastic Stable Intramedullary Nails (ESIN) are specifically designed to provide symmetrical elastic preloading within the medullary canal, generating balanced restoring forces and a predictable three-point fixation configuration [8,29]. This controlled elastic behavior promotes secondary bone healing through callus formation, rather than absolute rigidity. Therefore, the lower stiffness observed in the present study, particularly under torsional loading, is consistent with the load-sharing philosophy of flexible intramedullary fixation systems [6,26,27]. These characteristics should be considered when extrapolating the technique to feline fracture management, where implant selection must balance mechanical stability with a biological environment favorable to fracture healing.

The coefficient of variation was relatively high for both intact bones and constructs, likely due to natural variability in bone specimens. In the case of the constructs, additional variability may arise from differences in engagement with the endosteal surfaces and the proximal metaphyseal bone. This variability in construct stiffness may be clinically relevant, as constructs with lower stiffness may compromise proper fracture healing.

In the current study, maximum force values were high in axial compression tests, likely due to transverse fractures facilitating contact between the proximal and distal bone segments. However, the loading configuration cannot be considered purely axial, as force was applied through the femoral head, generating a bending moment [23]. Under these conditions, intact bones failed at the femoral neck, whereas stabilized fractures exhibited implant deformation and fracture-site disruption.

In the four-point bending tests, the constructs exhibited nail deformation without a distinct failure peak, whereas the intact bones fractured at the diaphysis. Therefore, bending behavior in constructs was described in terms of stiffness and deformation patterns rather than ultimate failure load. This progressive deformation pattern is characteristic of flexible fixation systems, which are designed to allow controlled elastic deformation under load rather than abrupt catastrophic failure. The bent ends of the flexible nails are anchored within the proximal cancellous metaphyseal bone [13,14], which is characterized by high porosity and low stiffness and may influence the deformation behavior observed in the tests. Additionally, the material properties of the flexible nails should be considered, as stainless steel demonstrates greater bending stiffness and generates higher elastic restoring forces than titanium nails of equivalent diameter [17].

Torsional testing showed low torque resistance, indicating a potential limitation of flexible nails in transverse fractures. A biomechanical study involving three-point bending and torsion tests on femurs from growing Beagles found that only one-third of spiral fractures stabilized with a flexible nail system achieved sufficient minimum stability, suggesting limited applicability for this fracture configuration [11]. On the other hand, these authors found it to be appropriate for stabilizing short oblique and transverse fractures.

Fractures stabilized with flexible nails may benefit from additional support provided by surrounding soft tissues, especially in closed femoral fractures [7,8], apparently independent of any specific force involved. These tissues limit excessive motion, while preservation of the periosteum supports biological healing [7]. When the appropriate nail diameter is selected, flexible nails allow controlled micromotions conducive to callus formation [7,8]. The relevance of these principles to cats remains to be further investigated.

The initial stiffness reduction point was calculated in axial compression and torsion tests to better approximate construct behavior under clinically relevant loading conditions. Study limitations include evaluation of only transverse fractures, absence of cyclic loading, and lack of comparison with alternative fixation systems. Other fracture patterns, including short oblique or more proximal/distal fractures, may yield different biomechanical results due to variations in endosteal engagement. Non-reducible fracture configurations are likely contraindications for this technique. In addition, the results may differ for fractures in more proximal or distal locations due to less engagement of the nails with the endosteum at those levels. Another limitation was the lack of comparison with other implant systems and the absence of cyclic testing.

## 5. Conclusions

In conclusion, flexible steel intramedullary nails with end caps demonstrate low construct stiffness, particularly under torsional loads, when used to stabilize mid-diaphyseal transverse femoral fractures in cats.

## Figures and Tables

**Table 1:** Descriptive statistics data of axial compression (Maximum Force, N), four-point bending (Maximum Force, N), and torsion (Maximum Torque, Nm) tests conducted on intact femurs of cats.

Tests	Mean ± SD	Standard Error	Coefficient Variation	Minimum	First Quartile	Median	Third Quartile	Maximum
Axial compression (n = 5)	1090.51 ± 374.12	167	34.31	683	703	1164	1441	1503
Four-point bending (n = 5)	1384.75 ± 191.08	85.4	13.79	1126.6	1214.5	1352.3	1571.7	1599.8
Torsion (n = 5)	6.76 ± 4.76	2.13	70.52	1.57	1.64	8.81	10.86	11.42

**Table 2:** Descriptive data of axial compression (Maximum Force, N), and torsion (Maximum Torque, Nm) tests conducted on femurs of cats with mid-diaphyseal transverse fracture stabilized with two flexible stainless-steel nails.

Tests	Mean ± SD	Standard Error	Coefficient Variation	Minimum	First Quartile	Median	Third Quartile	Maximum
Axial compression (n = 5)	608.43 ± 101.2	45.3	16.64	536.4	536.7	549.7	709.6	767.8
Torsion (n = 5)	0.166 ± 0.075	2.13	45.33	0.0820	0.0940	0.1600	0.2420	0.2580

**Table 3:** Values of stiffness (*P*/*δ*) for intact femurs and constructs (mid-diaphyseal transverse femoral fractures stabilized with two flexible stainless-steel nails) obtained through axial compression testing with eccentric load.

	Intact Bone		Construct		
No.	*P*/*δ* (*R*^2^) [N/m]	Range (Initial Displacement, Final Displacement) [mm]	No.	*P*/*δ* (*R*^2^) [N/m]	Range (Initial Displacement, Final Displacement) [mm]
1	1013.25 (0.9829)	(0.6, 0.8)	6	677.08 (0.9830)	(1.1, 1.3)
2	1078.02 (0.9795)	(0.6, 0.8)	7	118.46 (0.9921)	(0.35, 0.85)
3	676.50 (0.9986)	(0.3, 0.6)	8	110.18 (0.9001)	(0.35, 0.85)
4	832.29 (0.9687)	(0.6, 0.8)	9	499.25 (0.9618)	(1.0, 1.5)
5	629.81 (0.9933)	(0.6, 0.8)	10	89.31 (0.9968)	(0.35, 0.85)
Mean ± SD	845.98 ± 198.41		Mean ± SD	298.86 ± 271.69	

*P* = force, *δ* = displacement. Range = upper and lower crosshead displacement values used to calculate the linear stiffness.

**Table 4:** Values of stiffness (*P*/*δ*) for intact femurs and constructs (mid-diaphyseal transverse femoral fracture stabilized with two flexible stainless-steel nails) obtained through four-point bending tests.

	Intact Bone		Construct		
n^o^	P/δ (R^2^) [N/m]	Range (Initial Displacement, Final Displacement) [mm]	No.	P/δ (R^2^) [N/m]	Range (Initial Displacement, Final Displacement) [mm]
1	1480.20 (0.9984)	(0.1, 0.6)	1	12.02 (0.9928)	(1.2, 2.0)
2	1701.64 (0.9987)	(0.1, 0.6)	2	10.35 (0.9781)	(0.1, 0.6)
3	1554.56 (0.9977)	(0.1, 0.6)	3	12.61 (0.9607)	(0.1, 0.6)
4	1566.56 (0.9999)	(0.58, 0.86)	4	18.42 (0.9809)	(0.58, 0.86)
5	1597.65 (0.9964)	(0.1, 0.6)	5	13.20 (0.9909)	(0.67, 1.1)
Mean ± SD	1580.92 ± 72.23		Mean ± SD	13.32 ± 3.04	

*P* = force, *δ* = displacement. Range = upper and lower crosshead displacement values used to calculate stiffness.

**Table 5:** Values of torsion stiffness obtained through torsion testing in intact femurs and constructs (mid-diaphyseal transverse femoral fractures stabilized with two flexible stainless-steel nails).

	Intact Bone			Construct	
No.	*T*/*θ* (*R*^2^) [Nm/rad]	Range (Initial Displacement, Final Displacement) [rad]	No.	*T*/*θ* (*R*^2^) [Nm/rad]	Range (Initial Displacement, Final Displacement) [rad]
1	44.6013 (0.9982)	(0.0872, 0.174)	6	2.9947 (0.9614)	(1.134, 1.221)
2	10.9410 (0.9992)	(0.034, 0.069)	7	0.6737 (0.9989)	(2.042, 2.303)
3	30.3795 (0.9998)	(0.020, 0.061)	8	0.4966 (0.9901)	(0.663, 0.837)
4	43.6731 (0.9991)	(0.0872, 0.174)	9	1.0059 (0.9596)	(3.54, 3.595)
5	30.9353 (0.9998)	(0.0872, 0.174)	10	0.0368 (0.4442)	(0.645, 0.977)
Mean ± SD	32.11 ± 13.62 Nm/rad		Mean ± SD	1.04 ± 1.15 Nm/rad	

*T* = torque, *θ* = twisting angle, Nm/rad = newton meter per radian, [rad] = radians. Range = upper and lower crosshead displacement values used to calculate stiffness.

## Data Availability

The data that support the findings of this study are available from the corresponding author upon reasonable request.

## Abbreviations

The following abbreviations are used in this manuscript:

FIN	Flexible intramedullary nails
N	Newton
N/m	Newton per meter
Nm	Newton meter
Nm/rad	Newton meter per radian
mm	millimeter
°	degree
°C	degree Celsius
kN	kilonewton
$k_{t}$	torsion stiffness
*T*	applied torque
*θ*	twisting angle
SD	standard deviation
SE	standard error
CV	coefficient of variation
Q_1_	first quartile
Q_3_	third quartile
P/δ	stiffness parameter
